# The Week's Work

**Published:** 1911-07-01

**Authors:** 


### The Week's Work.

### THE NELSON HOSPITAL.

We understand that the foundation-stone of the new hospital for South Wimbledon, Merton, and district is to be laid on July 17 by the Duchess of Sutherland. As its name implies, the new hospital is being built as a memorial to England's most popular admiral, who often showed his interest in the sick and poor of Merton. Moreover, it was from Merton, we believe, that Nelson started for Trafalgar.

### ALTERATIONS AT ARBROATH INFIRMARY.

The Special Committee appointed in connection with the extension of Arbroath Infirmary, Forfar, N.B., has been supported by the directors in recommending Mr. Hugh Gavin, of Arbroath, to be architect, with Dr. Mackintosh, M.V.D., of the Western Infirmary, Glasgow, as consultant with him in carrying out the alterations. The cost of these, which will include new nurses' quarters, is expected to be £8,000, and an appeal for this sum is about to be issued. The present position of the hospital shows an accumulated debit of £276, with an increasing tale of patients. We note that ex-Provost Grant is again president.

### THE MIDWIFE IN AMERICA.

At the recent session the New York Academy of Medicine discussed the problem of the midwife, which has reached a stage there similar to that which existed in this country prior to the passing of the Midwives Act in 1902. The same morbidity and mortality, infantile as well as maternal, caused directly by the ignorance and uncleanliness of the midwives, have been demonstrated over there as prevailed also in Great Britain; and it is very interesting to note the lines along which it is proposed to remedy this very serious blot on the national polity and to remove the perpetual menace to the national health. Broadly speaking, the solution is being sought on much the same lines as our own; that is, by education and supervision of the midwives. In some matters of detail, however, it is the German model which is followed rather than our own. It is clearly recognised that a large section of the population must always rely rather on the midwife than on the doctor for attendance in childbirth, an economic proposition which is equally true in this country, and accounted for much that was and is distasteful to the profession in our own Midwives Act. The outlines of a scheme were proposed at the meeting aforesaid, including the establishment of schools for midwives in such existing outdoor maternity services as shall elect to do so; the establishment of similar training schools *de novo;* the provision in such maternity services of teachers especially qualified to train this class of pupil; and the provision of suitable textbooks, which apparently have yet to be written in the United States for midwives. Stress is laid on the point that the bulk of the instruction shall be practical and shall be given by paid instructors in the patient's own home, after a course of preliminary teaching in asepsis and of lectures on midwifery.

### HOSPITAL SUNDAY IN PARIS.

According to the "Standard," the French Red Cross Society, officially known as the "Union des Femmes Françaises," held a public "quête after the manner of the English Hospital Sunday." This, we learn, lead to the race and aeronautic meetings and theatres at night being thronged with beggars, who sold for two sous, and often more, on the "no change" principle, little badges, the wearing of which, however, protected the purchaser from further intimidation in the cause of charity. The official and unofficial arrondissements of Paris were divided between the questing bands; the Bois and the Halles were likewise infested; in short, the attempt to raise money in the French capital for the Red Cross Society seems to have shown a vigour that we hope will have been equally apparent when the total of our own Hospital Sunday collections is known.

### THE CENTRAL EYE HOSPITAL TO MOVE.

We are informed that, owing to the termination of its present lease, it has become necessary for the Committee of the Central London Ophthalmic Hospital to build a new hospital. A site near St. Pancras Station has now been secured. We understand that the proposed new building will cost £20,000 to complete, of which £12,000 already has been collected. This includes a grant of £1,000 from the King Edward's Hospital Fund, which, therefore, approves the scheme. Some £14,000 will be spent at once to construct a portion of the new buildings large enough to carry on the hospital's work. What we believe is virtually a first appeal to the public has been issued for the £8,000 necessary to complete the scheme. The foundation-stone of the new buildings in Judd Street, near St. Pancras Station, will be laid by the Duchess of Albany next Wednesday at 3 p.m.

### THE REBUILDING OF HOSPITALS AT LYONS.

At a recent meeting of the Académie de Médécine de Paris, M. Herriot, Mayor of Lyons, explained the plans of the new hospitals proposed to be erected by the municipality of which he is the representative. As a type of what it is intended to do in the matter of construction, he took the new Hôtel Dieu which is to be erected in the outskirts of Lyons on a site with a superficial area of sixteen hectares, or over thirty-two acres. This hospital is to have thirteen hundred beds, and all the University clinics, and is to be furnished with the latest hygienic and technical improvements. The following principles have been followed in elaborating the plans: The isolation of the pavilions, the separation in the interior of the surgical departments of septic from aseptic cases, the multiplication of isolation rooms for noisy and dying patients, the development of annexes such as eating and recreation rooms, galleries for taking the air, etc., modern installations for the application of new methods such as mecanotherapy, physiotherapy, hydrotherapy, permanent baths, etc., the extension of research and diagnostic laboratories, the isolation of the tuberculous, a special section for contagious cases with individual isolation carried out according to the methods of the Pasteur Institute; in short, all that makes for efficiency in a great modern hospital.

### THIS WEEK'S DRUG MARKET.

Since last week's report appeared business has naturally been at a standstill for several days owing to the Coronation holidays, and a quiet tone continued to prevail in the drug market during the earlier part of the present week. Consequently there is little of interest to record and price changes have been few in number. Citric acid is slightly dearer, as also is oil of almonds. Opium is unchanged in price. No further decline in the value of cod liver oil has taken place, but it is important to note that as a result of the better conditions that have lately prevailed in the fishing grounds, the yield of oil is now considerably larger than at the same date last year; it would therefore seem probable that lower prices for cod liver oil will be quoted in due course.

